# Supplementary figures and images for: Kinetics of the Cellular and Transcriptomic Response to Eimeria maxima in Relatively Resistant and Susceptible Chicken Lines
Source: Front Immunol. 2021 Mar 25;12:653085. doi: 10.3389/fimmu.2021.653085 (PMC8027475; doi:10.3389/fimmu.2021.653085)

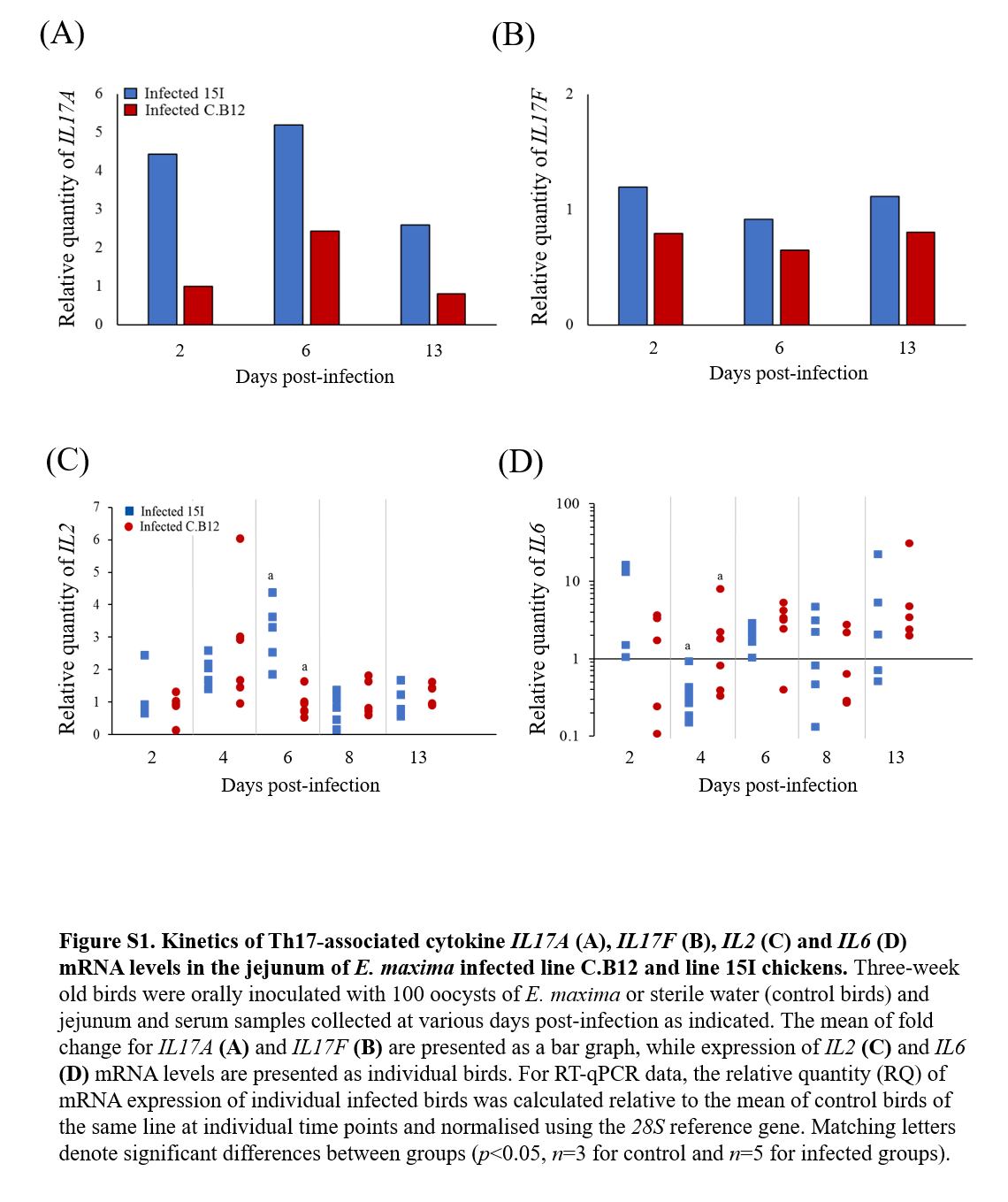

Supplement: Supplementary file 1 [file Image_1.tif]

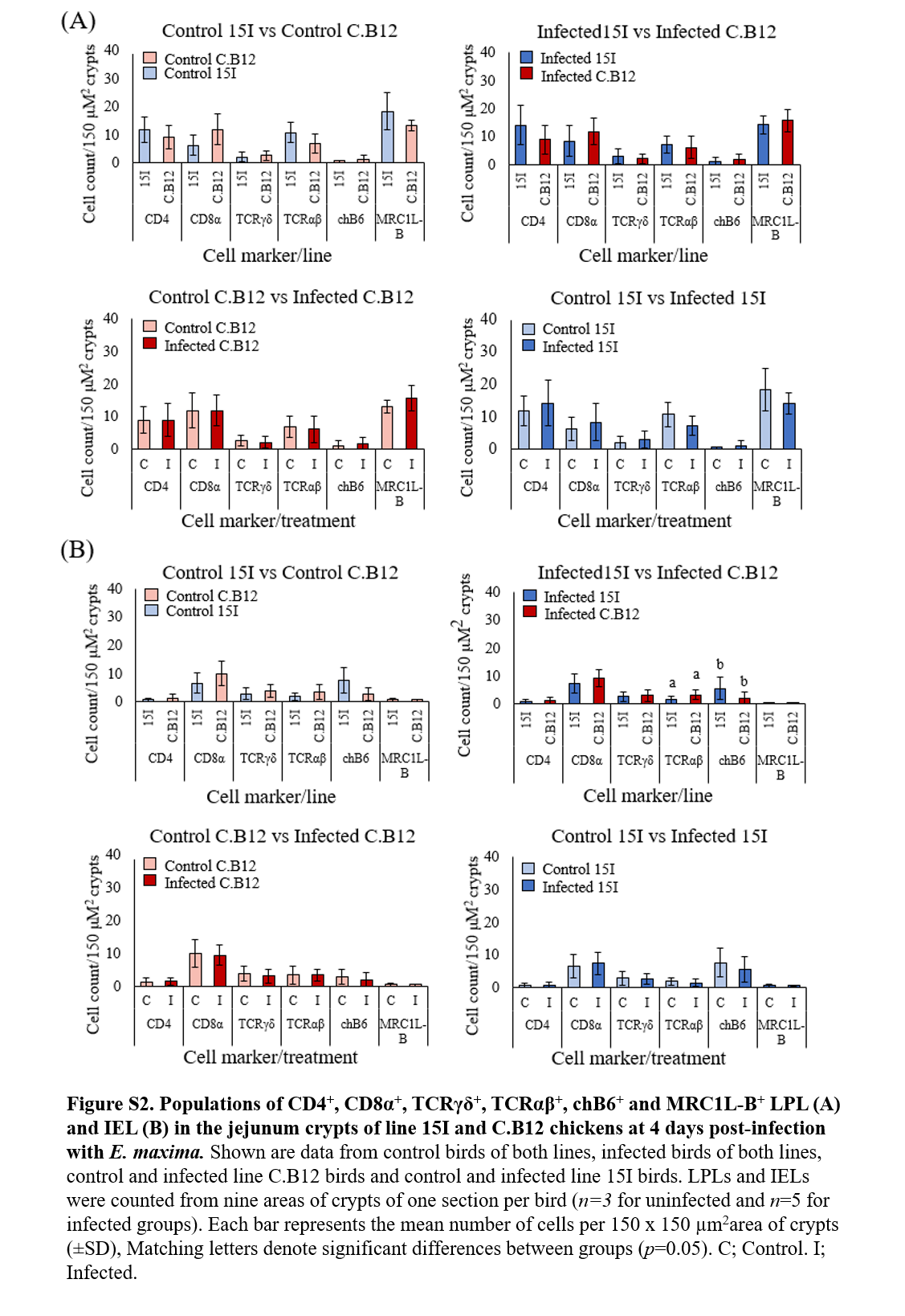

Supplement: Supplementary file 2 [file Image_2.tif]
